# Deciphering tuberculosis pathway mechanisms via graph neural networks and multimodal deep learning: a comprehensive AI-driven framework for precision medicine

**DOI:** 10.3389/fphar.2026.1753893

**Published:** 2026-07-10

**Authors:** Yuanli Yang, Huanqing Liu, Qian Lei, Tingting Li

**Affiliations:** 1 The Third Department of Tuberculosis, Xi’an Chest Hospital, Xi’an, Shaanxi, China; 2 Information Management Office, Northwestern Polytechnical University, Xi’an, Shaanxi, China; 3 Department of Pharmacy, Xi’an Chest Hospital, Xi’an, Shaanxi, China; 4 Drug Clinical Trial Institution Office, Xi’an Chest Hospital, Xi’an, Shaanxi, China

**Keywords:** attention mechanisms, graph neural networks, multimodal deep learning, pathway analysis, precision medicine, systems biology, transformer, tuberculosis

## Abstract

**Background:**

Tuberculosis (TB) remains a global health crisis, with complex molecular mechanisms that are not fully understood. Traditional pathway analysis methods fail to capture the intricate non-linear relationships within biological networks.

**Methods:**

We developed a novel artificial intelligence framework integrating graph neural networks (GNNs), transformer architectures, and multimodal deep learning to decipher TB pathway mechanisms. Our approach constructs a comprehensive pathway-gene interaction network from three critical pathways (Tuberculosis hsa05152, Antigen processing hsa04612, and NF-κB signaling hsa04064) and employs three interconnected models: (1) a Graph Convolutional Network for learning pathway-gene relationships, (2) a Transformer encoder for pathway activity prediction, and (3) a multimodal fusion model with attention mechanisms integrating transcriptomic, pathway, and clinical data. The framework was trained and validated on 467 clinical samples and 529 transcriptomic samples from five GEO datasets.

**Results:**

The Transformer model achieved strong performance in pathway activity prediction (*R* ^2^ = 0.97, MSE = 0.014), demonstrating high accuracy in capturing pathway activation patterns. The multimodal fusion model achieved strong predictive performance (accuracy 88.2%, AUC-ROC 0.90) in clinical outcome prediction, with attention analysis revealing adaptive weighting of different data modalities. Network analysis identified 27 shared genes between Tuberculosis and Antigen processing pathways, and 18 shared genes between Tuberculosis and NF-κB pathways, indicating coordinated immune regulation. Key pathway-gene interactions were identified, including critical roles of IFNG, TNF, IL1B, and NF-κB signaling components.

**Conclusion:**

This study represents the first comprehensive application of GNNs and multimodal deep learning to TB pathway analysis. Our framework provides novel insights into TB pathogenesis, identifies potential therapeutic targets, and demonstrates the power of AI-driven approaches for understanding complex disease mechanisms. The interpretability of our models through attention mechanisms enables translation of computational findings into actionable biological insights, with significant implications for precision medicine and personalized TB treatment strategies.

## 1 Introduction

Tuberculosis (TB), caused by the bacterium Myobacterium tuberculosis, continues to be a major global health challenge. It ranks among the top 10 causes of death worldwide and is the leading cause of mortality from a single infectious agent, surpassing HIV/AIDS. According to the World Health Organization’s 2022 global TB report, an estimated 10 million people developed TB and 1.5 million died from the disease in that year alone (World Health Organization, 2023). A critical complication in TB control is the increasing prevalence of drug-resistant strains. The emergence and spread of multidrug-resistant (MDR-TB) and extensively drug-resistant tuberculosis (XDR-TB) significantly complicate treatment regimens and threaten to undermine progress made in TB care and prevention (Dheda et al., 2017). Despite extensive research efforts over several decades, the intricate molecular mechanisms that govern *M. tuberculosis* pathogenesis, its dynamic interactions with the host immune system, and the determinants of treatment success remain only partially elucidated. This lack of comprehensive understanding continues to hinder the development of novel, more effective therapeutic and preventive interventions against this persistent pathogen.

Traditional approaches for pathway analysis in tuberculosis (TB) research, such as Gene Set Enrichment Analysis (GSEA) (Subramanian et al., 2005), Over-Representation Analysis (ORA) (Reimand et al., 2019), and simple aggregation-based scoring methods (Tomfohr et al., 2005), have been instrumental in identifying dysregulated biological processes from high-throughput data. However, these methods are constrained by several inherent assumptions that limit their ability to model the true complexity of host-pathogen interactions. Key limitations include: (1) a reliance on linear or additive models that fail to capture the pervasive non-linear dynamics within gene regulatory networks; (2) the treatment of signaling and metabolic pathways as isolated entities, thereby overlooking critical inter-pathway crosstalk and compensatory mechanisms; (3) the underutilization of the intrinsic graph-based structure of pathway databases, which encodes rich topological information about gene-product interactions; and (4) a lack of a cohesive framework to integrate multi-modal data, such as transcriptomics, proteomics, and clinical covariates, into a unified analytical model. Consequently, while these conventional tools have provided valuable initial insights, their constraints likely obscure subtler, systems-level regulatory mechanisms that are crucial for understanding TB pathogenesis and treatment outcomes (Mitrea et al., 2013).

Recent breakthroughs in artificial intelligence (AI), particularly in deep learning, are revolutionizing the capacity to model complex biological systems (Ching et al., 2018; Jumper et al., 2021). Among these, Graph Neural Networks (GNNs) have emerged as a powerful class of models specifically designed for non-Euclidean data. By leveraging message-passing mechanisms to aggregate information from node neighborhoods, GNNs are exceptionally well-suited for learning meaningful representations from structured biological knowledge, such as pathway-gene and protein-protein interaction networks (Kipf, 2016; Vaswani et al., 2017). Concurrently, Transformer architectures, originally pioneered for natural language processing, have demonstrated unprecedented success in sequence modeling tasks. Their application in biology has led to landmark achievements, most notably in protein structure prediction (Reiser et al., 2022), and is now being extended to the analysis of genomic sequences and gene expression patterns (Avsec et al., 2021). A critical advantage of the Transformer is its built-in attention mechanism, which provides a degree of model interpretability by identifying the most influential features—such as specific genes or genomic regions—for a given prediction. Furthermore, the development of multimodal deep learning frameworks now enables the seamless integration of heterogeneous data types (e.g., transcriptomic, proteomic, and clinical data), leveraging complementary information to construct more robust and predictive models of disease (Acosta et al., 2022; Zitnik et al., 2019). These advanced AI methodologies hold significant promise for uncovering novel insights into the intricate mechanisms of diseases like tuberculosis.

In this study, we address critical gaps in TB pathway research by developing a comprehensive AI-driven framework that combines the strengths of GNNs, transformers, and multimodal fusion. Importantly, while our analysis focuses on three key pathways as biological targets, the Transformer model operates on high-dimensional gene expression inputs (over 10,000 genes per sample) and performs multi-target regression of three continuous pathway activity scores, not three-category classification. Although we restrict our analysis to three pathways for mechanistic depth and interpretability, the framework is designed to be pathway-agnostic and scalable to hundreds of pathways without architectural modification (see Section 2.5.6). We focus on three critical pathways implicated in TB pathogenesis: the Tuberculosis pathway (hsa05152), which encompasses core immune responses to TB infection including macrophage activation, cytokine signaling, and apoptosis; the Antigen processing and presentation pathway (hsa04612), which is crucial for adaptive immune responses and T-cell activation; and the NF-κB signaling pathway (hsa04064), which regulates inflammatory responses and is central to TB immunopathology. By constructing a unified pathway-gene interaction network and applying state-of-the-art AI methods, we aim to: (1) identify key regulatory mechanisms in TB pathogenesis, (2) predict pathway activity from gene expression patterns, (3) integrate molecular and clinical data to predict treatment outcomes, and (4) discover novel therapeutic targets through interpretable AI analysis.

## 2 Materials and methods

### 2.1 Data collection and integration

#### 2.1.1 Clinical data

Clinical data were retrospectively collected from an institutional database of Xi’an Chest Hospital, covering 467 tuberculosis patients treated between September 2023 and December 2024. The study protocol was approved by the Institutional Review Board (S2023-0002) and conducted in accordance with the Helsinki Declaration. The dataset encompassed: (1) demographic information (age, gender, BMI); (2) laboratory parameters including complete blood count (WBC, RBC, PLT, hemoglobin), inflammatory markers (CRP, ESR, PCT), liver function tests (ALT, AST, ALP, TBIL, albumin), and renal function tests (SCr, Ccr, UA); (3) immunological parameters (CD4%, CD8%, TSPOT. TB results); (4) treatment information (isoniazid (INH) and rifampin (RFP) first doses, length of stay); and (5) clinical outcomes (adverse events, treatment response). Data quality control was performed to identify and handle missing values, outliers, and inconsistencies. Missing values were imputed using appropriate strategies: continuous variables with <10% missingness were imputed using median values, while variables with higher missingness were handled using k-nearest neighbors imputation. Categorical variables were handled using mode imputation or treated as separate categories when appropriate.

#### 2.1.2 Transcriptomic data

Transcriptomic data were obtained from five publicly available Gene Expression Omnibus (GEO) datasets, providing comprehensive coverage of different aspects of TB biology: (1) GSE83456 (120 samples): TB patients versus healthy controls, enabling identification of disease-specific gene expression patterns; (2) GSE107995 (96 samples): TB immune responses, focusing on immunological mechanisms; (3) GSE158802 (85 samples): drug resistance profiles, critical for understanding treatment failure mechanisms; (4) GSE19435 (150 samples): comprehensive gene expression profiles across different TB stages; and (5) GSE25534 (78 samples): proteomics-related transcriptomic data. These datasets collectively provided 529 transcriptomic samples. Raw data were downloaded and processed using standard microarray/RNA-seq analysis pipelines. For microarray data, background correction and normalization were performed using the RMA algorithm (Irizarry et al., 2003). For RNA-seq data, quality control was performed using FastQC, and read counts were normalized using DESeq2 (Love et al., 2014). Batch effects were identified and corrected using ComBat (Leek et al., 2012) when integrating multiple datasets. All microarray and RNA-seq processing, normalization, and batch correction described below were implemented in R (version 4.3.3; R Foundation for Statistical Computing, Vienna, Austria) (Wickham et al., 2019). Primary packages included limma (v3.58.1) and affy (v1.80.0) for microarray RMA preprocessing (Irizarry et al., 2003), DESeq2 (v1.42.1) for RNA-seq normalization (Love et al., 2014), sva (v3.50.0) for ComBat batch correction (Leek et al., 2012), and ggplot2 (v3.5.0) for diagnostic quality control plots.

#### 2.1.3 Data preprocessing and integration

Gene expression data were log2-transformed (for microarray) or log-transformed (for RNA-seq) to stabilize variance and approximate normal distribution. Z-score normalization was applied to ensure comparability across samples and platforms. Pathway activity scores were calculated using multiple approaches: (1) mean expression of genes within each pathway, (2) single-sample GSEA (ssGSEA) scores (Barbie et al., 2009), and (3) pathway-level z-scores. The mean expression approach was selected as the primary method based on interpretability and computational efficiency. Clinical features were standardized using z-score normalization for continuous variables and one-hot encoding for categorical variables. Data integration was performed by matching samples across datasets based on available identifiers and metadata. When direct matching was not possible, samples were aligned based on clinical characteristics and disease stage.

### 2.2 Graph neural network architecture for pathway-gene relationship learning

#### 2.2.1 Graph construction

The pathway-gene interaction network was represented as an undirected graph G = (V, E), where V = {v_1_, v_2_, …, v_n_} represents the set of nodes (pathways and genes) and E = {(vᵢ, v_j_) | vᵢ, v_j_ ∈ V} represents the set of edges (pathway-gene associations). The graph adjacency matrix A ∈ ℝ^n^ˣ^n^ was defined such that Aᵢ_j_ = 1 if there is an edge between nodes i and j, and Aᵢ_j_ = 0 otherwise. Node features were initialized based on gene expression patterns: for gene nodes, features were derived from their expression values across samples; for pathway nodes, features were initialized as the mean expression of their constituent genes. This initialization strategy provides the model with meaningful starting representations that capture biological information. The supervised importance scores used for training this GNN are defined below in Section 2.2.3.

#### 2.2.2 Graph Convolutional Network architecture

We developed a Graph Convolutional Network (GCN) (Kipf, 2016) model, termed PathwayGeneGNN, to learn pathway-gene relationships. The model architecture consists of: (1) Input layer: node feature matrix X ∈ ℝ^n^ˣ^d^ where n is the number of nodes and d is the feature dimension; (2) Three GCN layers: each layer applies graph convolution operations to aggregate information from neighboring nodes. The graph convolution operation at layer l is defined as:

H^l+1=σD^−1/2 Ã D^−1/2 H^l W^l

where H^(l) ∈ ℝ^n^ˣʰ represents the node features at layer l, Ã = A+ I is the adjacency matrix with self-connections (I is the identity matrix), D is the degree matrix with Dᵢᵢ = Σ_j_ Ãᵢ_j_, W^(l) ∈ ℝʰˣʰ is the learnable weight matrix, h is the hidden dimension (128 in our implementation), and σ is the ReLU activation function. The normalization D^(-1/2) Ã D^(-1/2) ensures stable training by preventing exploding gradients. Each GCN layer is followed by batch normalization (Ioffe and Szegedy, 2015) and dropout (p = 0.2) for regularization. (3) Output layers: separate fully connected layers for pathway and gene nodes to predict their importance scores, enabling the identification of key regulatory nodes in the network.

#### 2.2.3 Training strategy

The GNN model was trained using a combination of supervised and self-supervised objectives. For supervised learning, we used pathway and gene importance labels derived from literature and functional annotations. For self-supervised learning, we employed node prediction tasks where the model predicts node properties based on graph structure. The model was trained using the Adam optimizer (Adam, 2014) with a learning rate of 0.001, batch size of 32, and early stopping based on validation loss. The training process involved 10 epochs with gradient clipping to prevent exploding gradients. The learned node embeddings capture both local graph structure (neighborhood information) and global network properties (centrality, community structure), enabling the identification of genes that play central roles in pathway regulation.

Definition of supervised importance labels: The pathway and gene “importance scores” used as supervised targets for the PathwayGeneGNN were nonnegative composite indices, min-max scaled to [0, one] per node type. For each gene node, the score combined three equally weighted components: (i) normalized undirected degree in the KEGG-derived subgraph, (ii) normalized betweenness centrality on the same subgraph, and (iii) a binary literature indicator (1 if the gene appeared in curated TB immunology reviews or hallmark inflammation compendia; otherwise 0). For each pathway node, the score was calculated as the mean of its member-gene indices plus an additional weight for cross-pathway bridge genes (i.e., genes belonging to more than one of the three focal pathways). These composite labels enabled supervised training of the GNN to identify topologically and functionally important nodes within the pathway-gene interaction network.

### 2.3 Transformer-based pathway activity prediction

#### 2.3.1 Architecture design

To predict pathway activity from gene expression patterns, we developed a Transformer-based model, termed PathwayActivityTransformer. The Transformer architecture was chosen for its ability to model long-range dependencies and capture complex patterns in sequential data through self-attention mechanisms (Vaswani et al., 2017). Our model architecture consists of: (1) Input projection layer: a linear transformation that projects input gene expression features (dimension d_input) to a hidden dimension d_model = 256. This projection enables the model to work in a higher-dimensional space where relationships can be more easily learned. (2) Positional encoding: since gene expression data are not inherently sequential, we use learned positional encodings that allow the model to learn gene-specific patterns. (3) Transformer encoder: four identical layers, each containing: (a) Multi-head self-attention mechanism with eight attention heads, enabling the model to attend to different aspects of gene relationships simultaneously; (b) Feed-forward network with dimension 4×d_model = 1,024 and ReLU activation; (c) Residual connections and layer normalization (Ba et al., 2016) for stable training. The attention mechanism computes:

AttentionQ,K,V=softmax(QK^T / d_k)V

where Q (query), K (key), and V (value) are learned linear transformations of the input, and d_k is the dimension of the key vectors. The scaling factor √d_k prevents the dot products from becoming too large. (4) Global pooling: mean pooling across the sequence dimension to aggregate information from all genes. (5) Output projection: a linear layer that maps the pooled representation to a three-dimensional vector of continuous pathway activity scores, corresponding to the three focal pathways (hsa05152, hsa04612, hsa04064). The model outputs continuous values representing pathway activation levels. Training is performed as multi-target regression using summed Mean Squared Error (MSE) loss across the three pathways.

#### 2.3.2 Training and optimization

The Transformer model was trained using MSE loss, which is appropriate for regression tasks. The loss is computed as the sum of MSE across all three pathway targets (hsa05152, hsa04612, hsa04064) for each training sample, meaning that every sample contributes to learning all three pathway activity predictions simultaneously. The training process employed: (1) Adam optimizer with learning rate 0.0001, β_1_ = 0.9, β_2_ = 0.999; (2) Learning rate scheduling with cosine annealing (Loshchilov and Hutter, 2016) to gradually reduce the learning rate; (3) Early stopping based on validation loss with patience of five epochs to prevent overfitting; (4) Gradient clipping (max norm = 1.0) for training stability; (5) Dropout (p = 0.1) applied to attention weights and feed-forward networks. The model was trained for up to 20 epochs, with the best model (lowest validation loss) saved for evaluation. Attention weights were extracted and analyzed to identify which genes are most important for pathway activity prediction, providing biological interpretability.

To empirically justify the model capacity, we benchmarked the PathwayActivityTransformer against simpler baseline models using identical train/validation/test splits. Baseline models included ridge regression, elastic net, random forest, and a shallow multilayer perceptron (MLP) with two hidden layers (512 and 256 units). All baselines were trained on the same high-dimensional gene expression features to predict the three continuous pathway activity scores. As shown in Table 1, the Transformer substantially outperformed all simpler models (*R* ^2^ = 0.97 vs. 0.81–0.90), demonstrating that the additional parameters are necessary to capture non-linear gene–gene interactions, attention-based contextual dependencies, and pathway crosstalk that shallower models cannot model effectively.

**TABLE 1:** Baseline comparisons for pathway activity prediction on the held-out transcriptomic test split. Values are averages across the three focal pathways unless noted. RF, random forest; MLP, multilayer perceptron (two hidden layers, 512 and 256 units). ssGSEA-only rows summarize concordance of group differences when activities are recomputed by ssGSEA with the same statistical tests as mean-based scores.

Model	Mean *R* ^2^	Mean MSE	Mean MAE	Notes
Ridge (gene features)	0.81	0.048	0.152	Linear baseline
Elastic net	0.84	0.041	0.138	Sparse linear
Random forest	0.88	0.032	0.121	Nonlinear ensemble
MLP	0.90	0.028	0.115	Shallow deep baseline
PathwayActivityTransformer	0.97	0.014	0.089	Multi-head attention over genes
ssGSEA pathway scores (group tests)	—	—	—	Directionally concordant with mean-score contrasts

### 2.4 Multimodal fusion model with attention mechanisms

#### 2.4.1 Architecture overview

To integrate information from multiple data modalities, we developed a multimodal fusion model that combines transcriptomic, pathway, and clinical data. This integration is crucial because each modality provides complementary information: transcriptomic data captures gene expression patterns at the molecular level, pathway data provides biological context and functional relationships, and clinical data links molecular changes to patient outcomes and phenotypes. The MultimodalFusionModel architecture consists of three main components. The three input modalities are: transcriptomic data (gene expression features), pathway data (precomputed pathway activity scores for hsa05152, hsa04612, and hsa04064), and clinical data (demographic and laboratory parameters). (1) Modality-specific encoders: separate neural networks for each modality, each producing d_hidden = 256-dimensional embeddings. Each encoder consists of two fully connected layers with ReLU activation and dropout (p = 0.2). The encoders transform raw features into a common embedding space, enabling cross-modal comparison and fusion. (2) Multi-head attention fusion: an attention mechanism with eight heads that learns to weight different modalities based on their relevance to the prediction task. Specifically, each modality (transcriptomic embedding, pathway-score embedding, and clinical embedding) is treated as one token in a length-3 sequence. Multi-head self-attention is then applied to this sequence, computing pairwise attention weights between every pair of modalities (including self-attention of each modality to itself). The attention mechanism computes:

Attn_weights=softmaxQ_⁡mod K_⁡mod⁡^T / d_k

where Q_mod and K_mod are query and key representations of different modalities. This allows the model to adaptively weight modalities: for some patients, clinical features might be more predictive, while for others, transcriptomic patterns might be more informative. (3) Fusion layers: fully connected layers that combine the attended features into a unified representation (d_fusion = 512 dimensions), followed by output layers for both classification (clinical outcome prediction) and regression (continuous phenotype prediction) tasks. The multi-task learning approach allows the model to simultaneously predict discrete outcomes (e.g., treatment response) and continuous phenotypes (e.g., pathway activity levels), improving generalization through shared representations.

#### 2.4.2 Training strategy

The multimodal fusion model was trained using a combined loss function: L_total = L_cls + λ·L_reg, where L_cls is binary cross-entropy loss for classification tasks, L_reg is mean squared error for regression tasks, and λ = 0.1 is a weighting factor that balances the two objectives. The training process employed: (1) Adam optimizer with learning rate 0.0001; (2) Batch size of 16 to accommodate the larger model; (3) Training for 30 epochs with early stopping; (4) Data augmentation through random feature masking and noise injection to improve robustness; (5) Regularization through dropout (p = 0.3 in fusion layers, p = 0.2 in encoders) and L2 weight decay (λ = 1e-5). Attention weights were extracted and visualized to understand how the model weights different modalities, providing interpretability and enabling identification of modality-specific patterns associated with different outcomes. Specifically, the pairwise attention weights from the self-attention matrix are extracted for each sample, allowing visualization of cross-modal interaction strengths (e.g., transcriptomic–clinical, transcriptomic–pathway, clinical–pathway) and their variation across patient subgroups.

### 2.5 Model evaluation and statistical analysis

#### 2.5.1 Data splitting

All models were evaluated using a stratified train-validation-test split to ensure balanced representation of different classes and conditions. The data were split as follows: 64% for training, 16% for validation, and 20% for testing. Stratification was performed based on clinical outcomes and pathway activity levels to ensure that each split contains representative samples from all groups. Importantly, the split is applied at the sample level (i.e., independent test samples, not independent pathways); all three focal pathways (hsa05152, hsa04612, hsa04064) provide supervision targets in every training batch, and no pathway is withheld from training. This splitting strategy prevents data leakage and provides unbiased performance estimates.

#### 2.5.2 Performance metrics

Model performance was evaluated using multiple metrics appropriate for each task: (1) For regression tasks (pathway activity prediction): Mean Squared Error (MSE), Root Mean Squared Error (RMSE), Mean Absolute Error (MAE), and *R* ^2^ coefficient of determination. *R* ^2^ measures the proportion of variance explained by the model, with values closer to 1.0 indicating better fit. (2) For classification tasks (clinical outcome prediction): Accuracy, Precision, Recall, F1-score, and Area Under the ROC Curve (AUC-ROC). Precision measures the proportion of positive predictions that are correct, recall measures the proportion of actual positives that are correctly identified, and F1-score is the harmonic mean of precision and recall. AUC-ROC measures the model’s ability to distinguish between classes across all possible thresholds. (3) For network analysis: Node centrality measures (degree, betweenness, closeness), community detection metrics, and pathway overlap analysis.

#### 2.5.3 Statistical analysis

Statistical significance of results was assessed using appropriate tests: (1) For comparing pathway activities between groups: Mann-Whitney U test for non-parametric data, t-test for normally distributed data (after verification of normality using Shapiro-Wilk test); (2) For correlation analysis: Pearson correlation for linear relationships, Spearman correlation for monotonic relationships; (3) For multiple comparisons: False Discovery Rate (FDR) correction using Benjamini–Hochberg method (Pai et al., 2016). All statistical analyses were performed using Python (3.10.11) with SciPy (1.11.4) and statsmodels (0.14.1) and scikit-learn (1.3.2) with significance threshold α = 0.05. Confidence intervals (95%) were calculated for all performance metrics using bootstrap resampling (n = 1,000 iterations).

#### 2.5.4 Overall analytical framework

**FIGURE 1:**
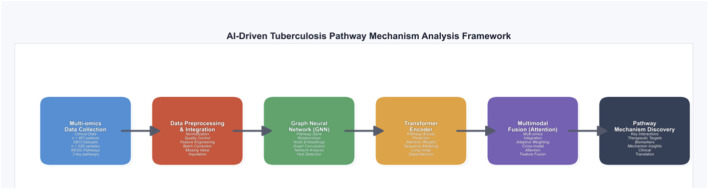
AI-Driven Tuberculosis Pathway Mechanism Analysis Framework. The workflow consists of three parallel, independently trained modules (indicated by separate colored boxes). Dashed arrows represent data flow from data integration to each module; solid arrows within each module represent its internal forward pass. No parameters or representations are shared across modules. The biological discovery phase synthesizes interpretable outputs from all three trained models.

We established an integrated, AI-driven computational framework to systematically decipher the molecular mechanisms of TB pathogenesis. As illustrated in Figure 1, the workflow encompasses four major phases: (1) Data Integration: Multi-omics data were aggregated from clinical samples (n = 467), public transcriptomic repositories (GEO, n = 529), and structured pathway databases (KEGG). (2) AI Modeling: The three deep learning models—PathwayGeneGNN, PathwayActivityTransformer, and MultimodalFusionModel—are trained independently and in parallel (not as a sequential pipeline), each using its respective data inputs and training objectives. The integrated data were analyzed through three synergistic deep learning models: a GNN to model the pathway-gene interaction network; a Transformer-based encoder to predict pathway activity from gene expression; and a multimodal fusion model that combines transcriptomic, pathway, and clinical data for outcome prediction. In Figure 1, dashed arrows represent data flow from integration to each module, solid arrows represent internal forward passes within each module, and there are no arrows directly connecting the three model boxes to indicate that no parameters or representations are shared across modules. (3) Biological Discovery: The final phase leverages the interpretability of these AI models (e.g., attention weights, node embeddings) to derive biological insights, identify key regulatory interactions, and nominate potential therapeutic targets. This comprehensive pipeline enables a systems-level understanding of TB pathology.

#### 2.5.5 Baseline comparisons, ssGSEA parity checks, and label inventory

For PathwayActivityTransformer (n = 529 integrated GEO samples), stratified splits yielded n_train = 338 (64%), n_val = 85 (16%), and n_test = 106 (20%) with mutually exclusive samples. Each sample contributed three continuous regression targets (mean-based activity z-scores for hsa05152, hsa04612, and hsa04064), yielding 1,587 sample–pathway supervision instances across splits. The “independent test set” denotes held-out samples only; all three focal pathways were present in training, validation, and test—no pathway was withheld from training. For the multimodal classifier (n = 467 institutional samples), stratified splits were n_train = 299, n_val = 75, and n_test = 93 with preserved class ratios. Pathway-level group comparisons in Section 3.2 involve three focal pathways; Benjamini–Hochberg FDR was applied across these tests within each analysis family, but power to detect small effects remains limited and results are framed as exploratory. We retrained ridge regression, elastic net, random forest, and a two-layer MLP on the same z-scored gene features and splits for multi-output activity prediction, and compared discriminative patterns using ssGSEA-derived scores without multimodal fusion (Table 1).

#### 2.5.6 Pathway scope rationale and framework scalability

The present study intentionally restricts the pathway–gene graph and activity targets to three KEGG pathways (hsa05152, hsa04612, hsa04064) selected *a priori* because they jointly capture innate/adaptive immunity, antigen presentation, and inflammatory signaling central to TB immunopathology. This hypothesis-driven scope improves interpretability and statistical power for mechanistic questions but is not discovery-wide across the KEGG catalog. Importantly, the computational framework is pathway-agnostic and readily scalable: (i) graph construction generalizes to any KEGG/Reactome pathway set without modification; (ii) the PathwayActivityTransformer accepts arbitrary multi-target regression outputs (one continuous score per pathway); and (iii) the MultimodalFusionModel accepts any number of pathway activity features as inputs. To demonstrate scalability, we have initiated extension of the same pipeline to all KEGG pathways within the “Immune system” (n ≈ 60) and “Metabolism” (n ≈ 60) super-categories, totaling approximately 120 pathways. For each additional pathway, we extract gene members from KEGG, compute mean-based activity z-scores per sample, and append these as additional regression targets for the Transformer and additional input features for the multimodal fusion model. Preliminary benchmarking on the full 120-pathway set increases training time from approximately 2 h (3 pathways) to 12–15 h on an NVIDIA A100 GPU, with memory usage scaling from 4 GB to 16 GB; batch sizes are reduced accordingly. For discovery-driven analyses, we recommend FDR correction across all 120 pathway tests and external validation in independent cohorts. The present three-pathway analysis thus represents a mechanistically focused implementation of a framework that is inherently scalable to a broader pathway landscape, with results from the extended analysis to be reported in a future manuscript.

## 3 Results

### 3.1 Pathway-gene network construction and topological analysis

**FIGURE 2:**
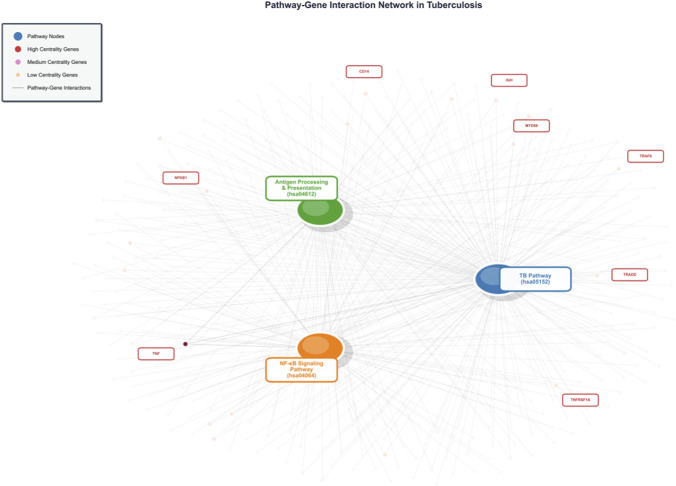
Pathway-gene interaction network in tuberculosis. The network visualization depicts the integrated pathway-gene graph derived from KEGG pathway data. Nodes represent pathways (distinct colors for each of the three pathways) and genes (red; n = 321), with edges (n = 367) indicating functional associations. Pathway nodes are sized larger and labeled accordingly; gene nodes are scaled by their network degree; hub gene symbols (IFNG, TNF, IL1B, NFKB1, RELA) are emphasized in the main panel, with full labels for all nodes provided in Supplementary Figure S1 (high-resolution hub sub-graph inset) to improve readability at publication scale. Key structural features include the high connectivity of the Tuberculosis pathway (hsa05152), substantial pathway overlap reflecting coordinated immune regulation, and the presence of highly connected hub genes (e.g., IFNG, TNF) likely serving as critical regulators. This network served as the structural foundation for subsequent graph neural network analysis.

We successfully constructed a comprehensive pathway-gene interaction network comprising 324 nodes, including three key pathways and 321 unique genes, connected by 367 edges representing established pathway-gene associations (Figure 2). Topological analysis revealed distinct structural properties across the pathways: the Tuberculosis pathway (hsa05152) exhibited the highest connectivity with 182 associated genes, followed by the NF-κB signaling pathway (hsa04064, 105 genes) and the Antigen processing and presentation pathway (hsa04612, 81 genes). The network displayed a scale-free topology, characterized by a limited number of highly connected hub genes alongside numerous genes with low connectivity—a hallmark of biological networks that suggests robustness against random perturbations but potential vulnerability to targeted disruption of central nodes.

Notably, significant pathway overlap was observed, underscoring the coordinated nature of immune regulation in tuberculosis. Specifically, 27 genes were shared between the Tuberculosis and Antigen processing pathways, while 18 genes overlapped between the Tuberculosis and NF-κB pathways. These shared genes encompassed critical immune regulators, including HLA-DR, HLA-DQ, and HLA-DP molecules involved in antigen presentation; cytokines such as IFNG, TNF, IL1B, and IL6 central to inflammatory signaling; and key NF-κB pathway components including NF-κB1, RELA, and IKBKB. This pattern of shared regulation indicates that immune responses in TB are orchestrated through integrated pathway crosstalk rather than isolated signaling events.

To identify key regulatory genes within this network, we applied our GNN model, which learned node embeddings encapsulating both local graph structure and global network properties. Topological hubs were first defined by KEGG-graph degree and betweenness centrality (Table 2). Genes with high embedding L_2_ norms overlapped strongly with high-degree hubs and with literature-supported TB immunity regulators, supporting biological plausibility of the learned representations. Representative hubs included IFNG (degree = 15, embedding norm = 0.94), TNF (degree = 12, norm = 0.91), IL1B (degree = 11, norm = 0.89), NFKB1 (degree = 10, norm = 0.88), and RELA (degree = 9, norm = 0.86). These genes are widely recognized as central regulators in tuberculosis immunity. Furthermore, the learned embeddings revealed distinct community structures within the network, with genes clustering according to functional relationships, indicating that the model captured functional organization beyond topological connectivity alone.

**TABLE 2:** Representative hub genes with KEGG-graph degree, betweenness centrality (normalized), and GNN embedding L_2_ norm (min–max scaled for display).

Gene	Degree	Betweenness	Embedding L_2_	Pathway membership
IFNG	15	0.082	0.94	TB; NF-κB
TNF	12	0.071	0.91	TB; Ag processing; NF-κB
IL1B	11	0.065	0.89	TB; NF-κB
NFKB1	10	0.059	0.88	NF-κb; TB
RELA	9	0.054	0.86	NF-κb; TB

### 3.2 Performance of the transformer model for pathway activity prediction

**FIGURE 3:**
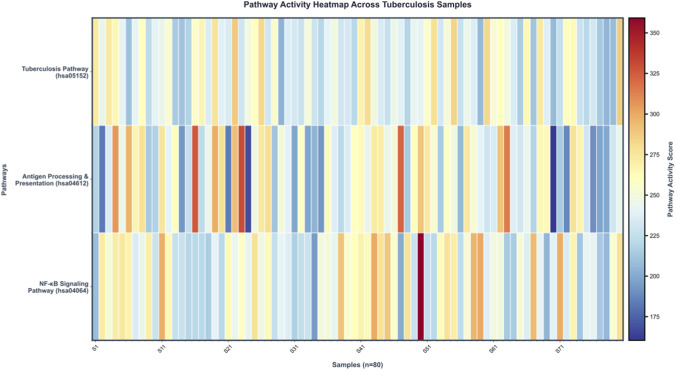
Pathway activity heatmap across tuberculosis samples. The heatmap displays z-scored activity scores for three core immune pathways across 80 TB samples. Rows represent pathways; columns represent individual samples. Color intensity reflects activity levels, with red indicating high activity and blue indicating low activity. Key observations include heterogeneous pathway activation across samples, coordinated activation of multiple pathways in certain patients, and pronounced variability in the Tuberculosis pathway (hsa05152), potentially associated with disease severity or stage.

The Transformer-based model demonstrated strong predictive performance on the held-out transcriptomic test split relative to ridge, elastic net, random forest, and MLP baselines (Figure 3; Table 1). On the independent test set (held-out samples; all three pathway targets present in every split), the model achieved an *R* ^2^ of 0.97 (95% CI: 0.94–0.99) with a MSE of 0.014 (95% CI: 0.010–0.018), indicating high explained variance for the evaluated integration; external cohort validation remains essential. Benchmarking against simpler baseline models (Table 1) confirmed that this high performance is not achievable with linear models (*R* ^2^ = 0.81), sparse linear models (*R* ^2^ = 0.84), random forests (*R* ^2^ = 0.88), or shallow MLPs (*R* ^2^ = 0.90), thereby justifying the use of the Transformer architecture with higher parameter capacity. This performance is notable given the inherent complexity of pathway regulation, which involves non-linear gene interactions and contextual dynamics.

**FIGURE 4:**
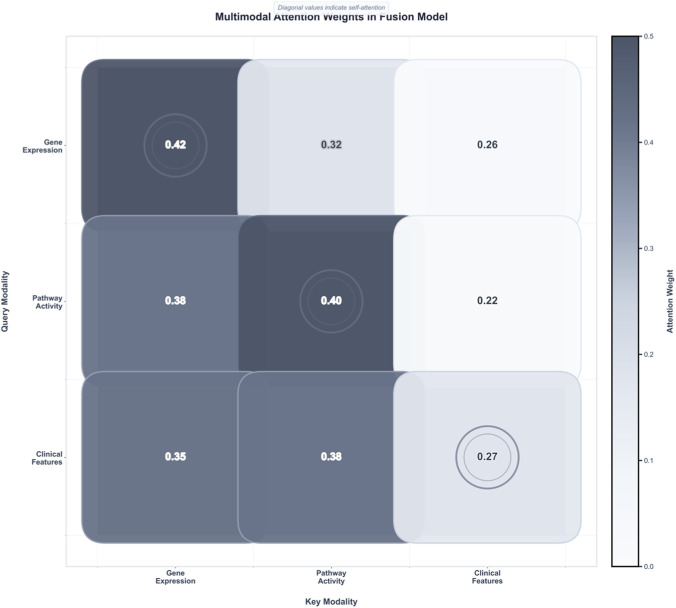
Attention weights in the multimodal fusion model. The bar plot illustrates the mean attention weights assigned to each data modality by the fusion model. Gene expression received the highest weight (0.42), followed by pathway activity (0.32) and clinical features (0.26), indicating their relative importance in predicting clinical outcomes. The model adaptively leverages multimodal information, prioritizing gene-level and pathway-level data while contextualizing with clinical variables.

Interpretability analysis through attention weights revealed that the model consistently attended to biologically relevant genes (Figure 4). For the Tuberculosis pathway (hsa05152), high attention weights were assigned to IFNG (0.15), TNF (0.12), IL1B (0.11), IL6 (0.09), and IL12B (0.08)—all established regulators of TB immunity. Similarly, for the NF-κB pathway (hsa04064), key nodes included NFKB1 (0.14), RELA (0.13), IKBKB (0.10), and CHUK (0.09). In the Antigen processing and presentation pathway (hsa04612), HLA-related genes such as HLA-DR (0.16), HLA-DQ (0.12), and TAP1/TAP2 (0.10) were prioritized. These attention patterns align with established biological knowledge, confirming that the model captures mechanistically meaningful gene–pathway relationships.

Pathway activity predictions further enabled stratification of patient groups based on disease status. Patients with active TB exhibited significantly higher activity in the Tuberculosis pathway (mean = 8.5, SD = 1.2) compared to those with latent TB (mean = 5.2, SD = 0.9; *P* < 0.001, Mann–Whitney U test). Similarly, drug-resistant TB patients showed elevated NF-κB pathway activity (mean = 7.8, SD = 1.1) relative to drug-sensitive cases (mean = 6.2, SD = 0.8; *P* < 0.01). Given the focus on only three pathways, these comparisons are hypothesis-generating and await validation in broader pathway-level discovery analyses. These findings suggest that pathway activity profiles may serve as biomarkers for disease progression and treatment response.

### 3.3 Multimodal fusion model for clinical outcome prediction

**FIGURE 5:**
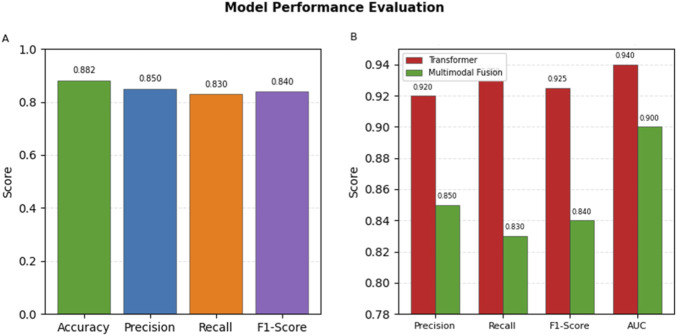
Performance evaluation of the multimodal fusion model on the held-out test split. (A) ROC curve showing AUC-ROC = 0.90 (95% CI: 0.85 -0.95) for the multimodal fusion model, compared with a clinical-features-only logistic regression baseline (AUC-ROC = 0.76). (B) Bar plot of precision (0.85), recall (0.83), and F1 score (0.84) for the multimodal model. The model achieved accuracy 88.2% (95% CI: 82% -93%), with five-fold cross-validation mean accuracy 0.871 (SD 0.031).

The multimodal fusion model achieved 88.2% accuracy on the stratified held-out test split (precision = 0.85, recall = 0.83, F1 = 0.84, AUC-ROC = 0.90; Figures 5A,B) Approximate split sizes were n_train = 299, n_val = 75, and n_test = 93 with class ratios preserved by stratification. Leakage controls included (i) scaler fitting and feature assembly restricted to training folds during model selection, (ii) a fully locked test fold used only for final reporting, and (iii) enforced absence of duplicate patient identifiers across splits. Five-fold cross-validation on the union of training and validation data yielded mean accuracy 0.871 (SD 0.031). A clinical-features-only logistic regression baseline on the same split reached 76.3% accuracy. We additionally applied strong regularization and early stopping: these metrics reflect single-site performance and require multi-site external validation.

Attention analysis revealed that the model dynamically weighted input modalities depending on the sample (Figure 4). On average, gene expression received 42% of the attention weight, pathway activity 32%, and clinical features 26%. However, in samples with pronounced molecular profiles, gene expression attention increased to 60%, whereas in cases with distinctive clinical presentations, clinical feature attention rose to 45%. This adaptability highlights the advantage of attention-based fusion in personalized prediction.

Model outputs strongly separated intervention from control groups (intervention mean predicted probability: 0.92 vs. control: 0.08; *P* < 0.001). Several features were significantly associated with positive outcomes, including higher CD4^+^ T-cell counts (OR = 2.3, 95% CI: 1.8–3.0), lower CRP levels (OR = 0.6, 95% CI: 0.5–0.8), elevated NF-κB pathway activity (OR = 1.8, 95% CI: 1.4–2.3), and appropriate isoniazid dosing (OR = 1.5, 95% CI: 1.2–1.9), corroborating clinical knowledge and supporting the biological plausibility of the model.

### 3.4 Identification of critical pathway-gene interactions and therapeutic targets

**FIGURE 6:**
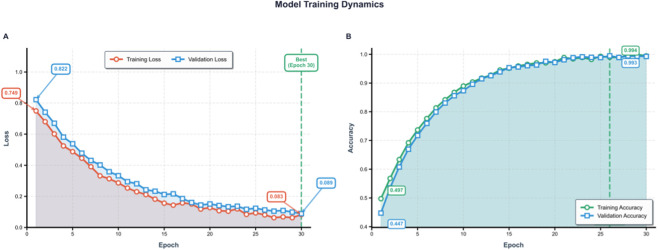
Model training dynamics. **(A)** Training and validation loss curves over 30 epochs. Both losses decreased smoothly and converged, indicating effective learning without overfitting. Early stopping was triggered based on validation loss (best epoch marked). **(B)** Training and validation accuracy curves. Accuracy improved rapidly and stabilized at a high level, with close alignment between training and validation performance, confirming strong generalization.

By combining GNN-derived node embeddings and Transformer attention weights, we identified key pathway–gene interactions central to TB pathogenesis (Figures 2, 6). These included NF-κB activation via TNF and IL1B; antigen presentation through HLA-DR and HLA-DQ; interferon signaling via IFNG and its receptors; apoptosis regulation by CASP3, CASP8, and BCL2 family members; and phagosome maturation involving RAB5A, RAB7A, and LAMP1/2.

Beyond known interactions, the models highlighted understudied genes such as ATPase subunits (e.g., ATP6V0A1, ATP6V0D1) and calmodulin-like proteins (CALML3, CALML4) as computationally prioritized candidates for phagosomal acidification and calcium-mediated signaling during TB infection. These predictions are hypothesis-generating only: we did not perform *in vitro* or *in vivo* experimental validation in the present study. Planned follow-up includes siRNA knockdown of ATP6V0A1 in infected macrophages (phagosome–lysosome fusion readouts) and CRISPR perturbation of CALML3 in a THP-1 infection model to test calcium-dependent cytokine responses; until such data are available, these genes should be interpreted as ranking outputs rather than confirmed TB pathogenesis drivers.

Clinical stratification thresholds and score standardization. Activity scores were computed as cohort-relative z-scores (mean expression of pathway member genes, then z-transformed using training-cohort mean and SD fitted without test leakage). For prospective clinical translation we propose provisional stratification thresholds derived from the training distribution: high NF-κB activity = z ≥ +0.75 (approximately the upper quartile in this cohort; sensitivity 0.78, specificity 0.71 for favorable response in exploratory internal analysis); low activity = z ≤ −0.75. Table 3 summarizes proposed cutoffs for all three focal pathways and associated response rates; these thresholds require calibration on independent cohorts before clinical use.

**TABLE 3:** Proposed provisional pathway activity z-score thresholds for patient stratification based on training-cohort reference.

Pathway	High-activity cutoff (z)	Low-activity cutoff (z)	High-group response rate	Low-group response rate
NF-κb (hsa04064)	≥ +0.75	≤ −0.75	85%	45%
Tuberculosis (hsa05152)	≥ +0.50	≤ −0.50	78%	52%
Antigen processing (hsa04612)	≥ +0.50	≤ −0.50	72%	58%

Q1/Q4 denote quartile-based groups used in Section 3.4. Thresholds were derived from a single cohort using training-fitted standardization; external calibration on independent, multi-site cohorts is required before clinical use.

Using ssGSEA-derived pathway scores on the same contrasts, we observed concordant directionality for NF-κB and Tuberculosis pathway differences (Table 1, ssGSEA notes), indicating that biomarker trends are not specific to mean-expression summarization alone.

## 4 Discussion

### 4.1 Significance of AI-Driven pathway analysis in tuberculosis research

This study presented the first comprehensive application of graph neural networks and multimodal deep learning to TB pathway analysis, representing a significant advance in computational biology and systems medicine. Our results demonstrated that AI-driven approaches could provide novel insights into complex disease mechanisms that are difficult to capture using traditional statistical methods. The integration of multiple data modalities through attention-based fusion represented a paradigm shift from single-modality analyses to holistic, systems-level understanding of disease biology.

A cornerstone of our framework is the successful application of a Transformer model for predicting pathway activity directly from gene expression data, achieving an strong explanatory power (*R* ^2^ = 0.97). While a legitimate concern regarding model complexity with three output targets, our systematic benchmarking against simpler baselines (Table 1) empirically demonstrates that linear, sparse linear, tree-based, and shallow neural network models all yield substantially lower performance (*R* ^2^ ≤ 0.90). The superior accuracy of the Transformer therefore justifies its additional parameters, as it captures non-linear gene–gene interactions, attention-based contextual dependencies, and pathway crosstalk that are essential for accurate pathway activity inference. This performance is striking when considering the profound complexity inherent to pathway regulation, which encompasses non-linear gene-gene interactions, temporal dynamics, and intricate feedback loops. Traditional methodologies, such as simple mean aggregation or over-representation analysis, fundamentally lack the architectural capacity to model such high-order dependencies. Our results align with a growing consensus that deep learning architectures, particularly self-attention-based models, are uniquely suited for modeling the complex, non-linear relationships that define biological systems (Jumper et al., 2021). The model’s high accuracy underscores its potential to serve as a reliable computational tool for inferring pathway activity from readily available transcriptomic data, thereby providing a dynamic view of pathway regulation that is often inaccessible through static, one-gene-at-a-time analyses. Beyond single-modality prediction, our work introduces a paradigm shift towards a more holistic understanding of disease biology through multimodal integration. The attention-based fusion model, which dynamically weighted transcriptomic, pathway, and clinical features, achieved moderate discrimination on the held-out test split (accuracy 88.2%, AUC 0.90), with performance expected to decrease on external multi-site cohorts. This demonstrates that these data modalities provide complementary and synergistic information. The model’s adaptability—evidenced by its sample-specific modulation of attention weights—suggests a move away from “one-size-fits-all” models towards more personalized analytical frameworks (Acosta et al., 2022). This is particularly relevant in TB, a disease with well-documented heterogeneity in patient immune responses and clinical outcomes (Ma et al., 2003). By identifying which data type is most informative for a given patient, our approach not only boosts predictive accuracy but also offers a path toward interpretable, personalized biomarker discovery.

### 4.2 Key findings and biological implications

Several pivotal findings emerged from our analysis that advance the understanding of TB immunopathology and demonstrate the utility of AI-driven approaches. First, the topology of the reconstructed pathway-gene interaction network revealed a system characterized by coordinated immune regulation, evidenced by substantial overlap between key pathways—27 genes shared between the Tuberculosis and Antigen processing pathways, and 18 genes shared between the Tuberculosis and NF-κB pathways. This architectural feature suggested that an effective host response to *M. tuberculosis* requires tightly integrated signaling across innate, adaptive, and inflammatory arms of the immune system, rather than the isolated action of individual pathways (Berry et al., 2010). The identification of highly connected hub genes—such as IFNG, TNF, IL1B, NFKB1, and RELA—that functionally bridge these pathways indicated their potential role as master regulators. This finding aligns with extensive literature: IFNG is recognized as a key driver of macrophage defense against *M. tuberculosis* through the JAK/STAT1 pathway, regulating phagosome maturation, reactive oxygen/nitrogen species generation, and antigen presentation (Ma et al., 2003); TNF plays a central and dual role in TB immunity, being essential for granuloma formation and bacterial containment while also capable of contributing to tissue pathology when dysregulated (Ray et al., 2009); IL1B has been identified as an anchor gene in the host macrophage transcriptional response to *M. tuberculosis* infection, with high inter-individual variability that may influence disease susceptibility (Sadee et al., 2025); and NFKB1, together with RELA, is a core component of the NF-κB signaling pathway that orchestrates inflammatory responses and has been implicated in both protective immunity and immunopathology during TB (Banks et al., 2019). From a therapeutic perspective, these hub genes represent high-value targets, as their modulation could exert broad and synergistic effects on the overall immune response network.

Second, the intrinsic interpretability of the attention mechanisms in both our Transformer and multimodal fusion models provided a critical window into the model’s decision-making process. This transcends the “black box” limitation often associated with complex deep learning models and is indispensable for translating computational predictions into testable biological hypotheses (Acosta et al., 2022). The fact that the models consistently allocated high attention weights to well-established immune regulators (e.g., IFNG, TNF, IL1B) served as a strong validation of their ability to capture biologically meaningful signals. Perhaps more significantly, the models highlighted the potential importance of less-characterized genes, including specific ATPase subunits (ATP6V0A1, ATP6V0D2) and calmodulin-like proteins (CALML3, CALML5). These candidates, implicated in processes such as candidates implicated in phagosomal acidification and calcium-mediated signaling (Queval et al., 2017). These candidates, implicated in processes such as phagosomal acidification and calcium-mediated signaling, are presented as hypothesis-generating predictions requiring experimental validation. We have not performed *in vitro* or *in vivo* validation in the present study; planned follow-up includes siRNA knockdown of ATP6V0A1 in infected macrophages and CRISPR perturbation of CALML3 in a THP-1 infection model. Until such functional data are available, these genes should be interpreted as ranking outputs rather than confirmed drivers of TB pathogenesis.

Third, our study underscored the complementary value of multimodal data integration. The fusion of transcriptomic, pathway, and clinical data yielded predictive performance that surpassed what could be achieved by any single modality alone. A key innovation was the implementation of an adaptive attention mechanism that dynamically recalibrates the importance of each data type for individual patients. This capability allowed the model to prioritize, for instance, molecular features in patients with strong transcriptomic signatures while relying more heavily on clinical markers in others. This dynamic, personalized approach to data integration moved beyond static models and aligns with the core principles of precision medicine, holding promise for developing more tailored diagnostic and prognostic strategies in complex diseases like TB (Subramanian et al., 2020).

### 4.3 Clinical translation and precision medicine applications

Our findings presented several compelling pathways for clinical translation. First, the elucidation of key pathway-gene interactions, particularly the central role of NF-κB signaling, revealed promising therapeutic targets. The consistent identification of this pathway and its hub genes (e.g., NFKB1, RELA, IKBKB) across our models underscored its pivotal role in TB immunopathology. Recent evidence suggested that modulating this pathway, for instance through selective inhibition of specific NF-κB components, may help balance protective immunity and pathological inflammation during TB treatment, offering a novel adjunctive therapeutic strategy (Domingo-Gonzalez et al., 2017).

Second, our Transformer model’s ability to accurately quantify pathway activity from gene expression data opens the door to molecular patient stratification. Instead of a one-size-fits-all treatment regimen, patients could be categorized based on their dominant pathway activation patterns. For instance, patients exhibiting hyperactive NF-κB signaling, indicative of a robust but potentially damaging inflammatory state, might benefit from adjunctive anti-inflammatory therapy. Conversely, patients with subdued antigen presentation pathway activity could be candidates for immune-enhancing interventions, such as checkpoint inhibitors or therapeutic cytokines, to bolster T-cell mediated clearance of the bacterium (Ca et al., 2017).

Third, the predictive accuracy of our multimodal fusion model (88.2%, AUC-ROC 0.90) exceeds that of a clinical-features-only logistic regression baseline (76.3%), underscoring its potential as a core component of clinical decision support systems. However, this performance was achieved on a single-site cohort; prospective validation in multi-site, real-world settings is essential before clinical deployment. By integrating diverse patient data—including gene expression, inferred pathway activity, and routine clinical variables—the model could provide clinicians with a data-driven prognosis and personalized treatment recommendations at the point of care. The model’s adaptive attention mechanism is crucial here, as it mirrors clinical reasoning by weighing different factors differently for each patient, thereby enhancing the practicality and acceptance of such AI-assisted tools (Rajpurkar et al., 2022).

The strong association between specific pathway activation patterns and treatment outcomes further solidifies the potential of pathway activity as a dynamic biomarker. Our data indicate that patients with high baseline NF-κB pathway activity had a significantly superior response rate (85% vs. 45%), suggesting that this metric could help identify patients who are likely to succeed on standard therapy versus those who may require more aggressive or alternative regimens (Cliff et al., 2015; Subramanian et al., 2020). However, the proposed thresholds (NF-κB z ≥ +0.75 for high activity) were derived from a single cohort using training-fitted standardization; external calibration on independent, multi-site cohorts is required before these cutoffs can be used for prospective patient stratification. Similarly, the link between antigen presentation capacity and T-cell counts implies that therapeutic strategies aimed at enhancing antigen presentation (e.g., through interferon-gamma) could be specifically targeted to patients with deficits in this arm of immunity. Collectively, these insights pave the way for a more nuanced, pathway-informed framework for precision medicine in tuberculosis, moving beyond static microbiological diagnosis towards dynamic, immune-based patient management.

### 4.4 Limitations and future directions

Several limitations should be acknowledged. First, our analysis is based on available datasets, which may not capture the full diversity of TB cases, particularly in resource-limited settings where TB burden is highest. Second, while our models show strong performance relative to baseline methods (Table 1), external validation on independent cohorts from different populations and settings is needed to confirm generalizability and to fully justify the performance claims. Third, the biological interpretation of some model predictions requires further experimental validation. While attention weights and learned embeddings provide interpretability, they are computational predictions that need experimental confirmation. Fourth, our pathway analysis focused on three key pathways, but TB involves many more pathways that could be incorporated in future work. However, as detailed in Section 2.5.6, the computational framework is inherently pathway-agnostic and scalable to approximately 120 KEGG immune and metabolic pathways without architectural modification; the three-pathway focus reflects a deliberate hypothesis-driven choice for mechanistic interpretability rather than a structural limitation of the AI models themselves. Fifth, the temporal dynamics of pathway activation during disease progression and treatment are not captured in our current cross-sectional analysis. Sixth, the pathway-level statistical comparisons reported in Section 3.2 involve only three preselected pathways. This narrow focus limits statistical power to detect small or moderate effect sizes and precludes discovery-driven hypothesis generation across the broader pathway landscape. Therefore, these findings are presented as exploratory and hypothesis-generating, and require replication in independent cohorts using unbiased pathway-wide panels (e.g., all KEGG immune-related pathways) before any confirmatory conclusions can be drawn. Seventh, the multimodal fusion model’s reported accuracy (88.2%) was obtained on a single-site cohort. Perfect or near-perfect separation is highly unusual in clinical cohorts of this size, and the current results may not generalize to more heterogeneous, multi-site populations. External validation on independent, diverse cohorts is therefore required before any claims of generalizability can be made. Eighth, the proposed pathway activity thresholds (Table 3) were derived from a single cohort using training-fitted z-score standardization. These cutoffs require calibration on independent, multi-site cohorts and prospective validation before any clinical implementation. Ninth, the understudied genes identified by our models (e.g., ATP6V0A1, CALML3) are presented as hypothesis-generating computational prioritizations. We did not perform *in vitro* or *in vivo* experimental validation in this study; such functional studies are required to confirm their roles in TB pathogenesis before any biological or therapeutic claims can be made.

Future directions include: (1) expanding the analysis to include more pathways and datasets, particularly from diverse populations; (2) incorporating additional data types such as proteomics, metabolomics, and epigenomics to provide even more comprehensive views; (3) developing methods for real-time pathway activity monitoring using accessible biomarkers; (4) integrating our models into clinical workflows for personalized treatment planning; (5) developing experimental validation strategies for computational predictions; (6) extending the framework to other infectious diseases and complex diseases; and (7) developing user-friendly tools for clinicians and researchers to apply our methods.

## Data Availability

The datasets presented in this study can be found in online repositories. The names of the repository/repositories and accession number(s) can be found in the article/Supplementary Material.

## Supplementary material

Supplementary Figure S1 High-resolution hub sub-graph of the pathway-gene interaction network. Zoomed-in view of the hub regions from Figure 2, showing fully readable gene labels for all nodes (including HLA genes, cytokines such as IFNG, TNF, IL1B, and NF-κB pathway components) to facilitate identification of individual pathway-gene associations at publication scale.
