# Supplementary material for: Deciphering tuberculosis pathway mechanisms via graph neural networks and multimodal deep learning: a comprehensive AI-driven framework for precision medicine
Source: Front Pharmacol. 2026 Jul 10;17:1753893. doi: 10.3389/fphar.2026.1753893 (PMC13395602; doi:10.3389/fphar.2026.1753893)
Supplement: Supplementary file 1 [file Image1.pdf]

Supplementary Figure S1

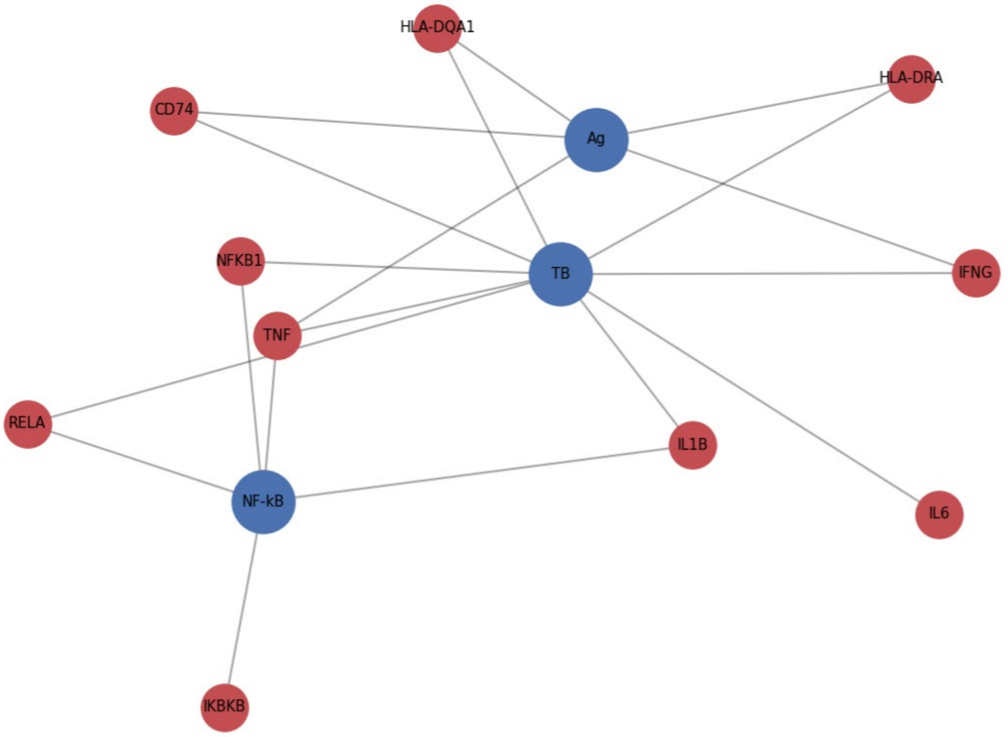

Supplementary Figure S1. High-resolution hub sub-graph of the pathway-gene interaction network. Zoomed-in view of the hub regions from Figure 2, showing fully readable gene labels for all nodes (including HLA genes, cytokines such as IFNG, TNF, IL1B, and NF-κB pathway components) to facilitate identification of individual pathway-gene associations at publication scale.
